# A rapid appraisal of the status of mental health support in post-rape care services in the Western Cape

**DOI:** 10.4102/sajpsychiatry.v23.959

**Published:** 2017-01-26

**Authors:** Naeemah Abrahams, Aník Gevers

**Affiliations:** 1 Gender and Health Research Unit, South African Medical Research Council, South Africa; 2 Independent Consultant, Honorary Faculty Adolescent Health Research Unit, University of Cape Town, South Africa

## Abstract

**Background:**

Despite the well-known impact of rape on mental health and the widespread problem of rape in South Africa, mental health services for rape victims are scant and not a priority for acute-phase services. Survivors encounter multiple mental health struggles in this period including adherence to the post-exposure prophylaxis drugs to prevent HIV and finding support from important others. We have little information on what mental health is provided, by whom and how it is integrated into the post-rape package of care.

**Aim:**

The aim of the study was to do a rapid appraisal of mental health services for rape survivors to gain a better understanding of the current acute and long-term (secondary) mental health services.

**Method:**

We conducted a qualitative study using a rapid assessment with a purposive sample of 14 rape survivors and 43 service providers recruited from post-rape sexual assault services in urban and rural Western Cape Province. Data were collected using semi-structured in-depth interviews and observations of survivor sessions with counsellors, nurses and doctors. The data were coded thematically for analysis.

**Results:**

Survivors of rape experienced a range of emotional difficulties and presented varying levels of distress and various levels of coping. Receiving support and care from others assisted them, but the poor integration of mental health within post-rape services meant few received formal mental health support or effective referrals. Multiple factors contributed to the poor integration: mental health was not given the same level of priority as other rape services (i.e. clinical care, including forensic management), the inadequate capacity of service providers to provide mental healthcare, including mental health illiteracy, the lack of continuity of care, the poor linkages to ongoing mental healthcare, and the mental health challenges caused by vicarious trauma and compassion fatigue.

**Conclusion:**

Providing effective, compassionate mental health services should be seen as essential components of post-rape care. The strengthening of support for providers and linkages to ongoing mental healthcare are essential to improve mental health services within acute post-rape services.

## Introduction

Sexual violence is a common trauma experience among women in South Africa.^1,2^ National prevalence studies have not been conducted; hence, rapes reported to police remain the main data source, albeit problematic. A total of 62 649 sexual offences were reported to the police for the 2013 and 2014 year, of which 73.8% (46 253) were rapes.^3^ This is an underestimate as under-reporting of rape to the police is a well-known phenomenon.^4,5^ The extent of rape in South Africa can also be gleaned from perpetration prevalence studies of men where between 28.0% and 37.0% of men disclosed rape perpetration. Studies of women found one in four reported experiences of sexual violence in their lifetime.^5,6,7^

Mental health challenge following sexual assault is a serious but under-recognised problem.^8,9,10^ The few studies conducted in South Africa have shown that rape had the strongest association with PTSD^1,11^ while work done by Abrahams and colleagues^2^ found depression symptomatology to be high 1 month after the rape with 62.8% of the women having evidence of depression and suicidality. Despite the well-known impact of rape on mental health^1,8^ and the widespread problem of rape in South Africa,^7^ mental health services for rape victims appear scant and not a priority for post-rape services.^12^ Progress in post-rape services has mainly been in the medico-legal response to rape through the increase in Thuthuzela Care Centres (TCCs).^13^ These centres are mainly urban based and attached to public hospitals and their main aims are to reduce secondary victimisation, increase convictions and reduce the time taken to finalise prosecution. The mental health support provided at the TCCs is largely by non-governmental organisations (NGOs) and has been critiqued recently as being a cursory response.^12^

The mental health impact of rape creates vulnerabilities for HIV infection both in the immediate post-rape period, during which many survivors take post-exposure prophylaxis (PEP), and in the long term. A study on adherence to the PEP drugs showed how prioritising HIV prevention and completion of the PEP medication interferes with survivors’ ability to adhere to the drugs.^14^ The study reported a complex array of reasons for poor adherence including fear of rape stigma, fear of HIV infection and poor support, which caused distress to the survivor and compromised their ability to take the drugs. This study’s conclusions highlighted the importance of psychosocial support in this period of taking PEP drugs but noted the limited information available on who provides mental health support, what is provided and how mental healthcare is delivered. The development of mental health support for rape victims immediately after rape requires a detailed understanding of what mental health services are available at the acute level of care, what possibilities there are for referrals and the nature of mental health needs after rape. This paper presents the findings of a rapid assessment done to describe and understand the mental health services and available referral pathways for rape victims at primary-level care so as to provide a basis from which to develop responsive services.

## Research methods

We conducted a qualitative study using a rapid assessment approach, including in-depth interviews with service providers and rape survivors as well as observations at rape centres.

### Study sites

We recruited participants from centres in urban (Cape Town Metropole District) and rural (Overberg District) Western Cape Province (see Table 1) from primary response services including TCCs^3^ and a Crisis Centre based at a hospital.^1^ Secondary response services offering long-term mental health support included nine NGOs, one secondary-level hospital and two private practices. We also included five sexual assault response staff from five police stations (see Table 1).

**TABLE 1:** Description of study sites and participants.

Site category	Site description	Participants
Acute-level response (acute, short-term)	3 Rape Care Centres (urban)	12 survivors (female) 3 medical doctors 1 nurse 3 victim assistance officers/site managers 6 lay counsellors
	1 Community Health Centre (CHC) (rural)	2 survivors (female) 2 nurses 1 lay counsellor
	5 SAPS Stations (4 urban; 1 rural)	6 police officers 1 police social worker 2 VEP volunteers
Secondary-level response (long-term)	9 NGOs (9 urban)	15 service providers (also filled roles of coordinators and managers)
	1 Secondary-level hospital	1 clinical psychologist
	2 Private Practice (urban)	2 clinical psychologists

All interviews were conducted in the language the participants were comfortable with. Three participants requested interviews be done in isiXhosa and an experienced research assistant fluent in isiXhosa assisted the author (AG) in these interviews.

### Participants

In total, 43 interviews were conducted with service providers including a range of healthcare professionals, sexual assault site managers, victim assistance officers, police officers and lay counsellors or volunteers.

The second sample included 14 adult rape survivors and we purposefully recruited women who were eligible for the 28-day PEP regimen as we wanted to determine the link between mental health service provision and the use and adherence to PEP medication. We stopped recruitment when we reached saturation at 14 participants.

### Observations and field notes

Detailed field notes were done as an additional data source. All the observations were conducted with the consent of the service provider and the rape survivors. Field notes were written to document the researchers’ observations of sessions between rape survivors and counsellors, nurses and doctors. This additional layer of data was found to be very useful as often the researcher had done or planned to do interviews with the same service provider being observed and the observation field notes of the interaction with rape survivors highlighted the subtle differences between the interview and the observation data. Field notes were also developed from the researcher’s observations while waiting with participants in the waiting room. Field notes were also recorded immediately after interviews and reflected the researcher’s own experiences and perceptions of the interviews and provided further context and clarifications of the interviews.

### Interview guides

Interview guides were developed at the proposal development stage but the qualitative nature of the study allowed for the introduction of discussions not initially identified at the planning stage. The guides were based on the literature around service provision for rape survivors, mental health after rape, support and training as well as the sexual assault policy developed by the Department of Health (DoH).^15^ Separate interview guides were developed for each set of service providers interviewed and this ensured the exploration and descriptions of their role in post-rape care. These included: their training to provide this service, their perspectives on PEP adherence and retention within health and other services, their perspectives of survivors’ mental health needs and mental health support within the service package, and their personal experiences of working in post-rape care. The latter was not in the development of the initial guide and was added after service providers discussed how working with rape survivors impacted on their personal and work lives as well as their own mental health.

We also developed an interview guide for rape survivors, and this was based on the literature, our experiences working with rape survivors and our experiences working in mental health services. The interview guide explored the post-rape services they received and how they accessed these services; their experiences of these services; long-term support services (if applicable) and interaction with various service providers; taking PEP medications; and their coping strategies and support needs throughout the post-rape period.

### Study rigour

Rigour was ensured by doing the interviews in the participants’ preferred language and transcribing the interviews verbatim. In addition, we were able to do triangulation with the multiple data sources from participants, service providers and field notes. Our study sites included both urban and rural services and from primary- and secondary-level services. Rigour was also ensured by the inclusion of a wide range of service providers with whom the rape survivor engages with at the time of reporting a rape and in post-rape care, that is, police victim support staff, lay counsellors, social workers, nurses, psychologists, forensic doctors and primary healthcare staff, which allowed for effective triangulation of the data. Finally, to ensure the credibility of the data, we had two peer debriefing meetings. One was with experts and the other with rape service providers. The latter meeting was to share the study outcomes and to ensure the findings were reflective of the service provider experiences.

### Data analysis

All interviews were audio recorded with the permission of participants. All interviews were transcribed and the three isiXhosa interviews were translated into English. We used the Framework Approach for analysis, which is commonly used in health research and in applied policy research.^16^ This approach uses a thematic analysis and we started analysis by familiarising ourselves with the data from the time of data collection, doing field notes, transcription process and the many readings of the interviews. This method allows for an a priori approach, which allows for broad themes from the interview guides in the initial coding process. To ensure rigor, both authors agreed on the themes that emerged in the coding and sub-coding process and we presented and discussed the thematic codes with a group of research colleagues and experts (Academic meetings). The observation data, recorded in researcher notes, were used to provide contextual understanding of the interview data and assisted in the coding process. We also considered the DoH Sexual Assault policy as a guide for the analysis of services experienced and provided.

### Ethical considerations

This study was granted ethical approval by the South African Medical Research Council. In addition, permission was granted to conduct this study in the acute service sites by the Western Cape DoH, the National Prosecuting Authority (NPA), and the provincial commissioner for the South African Police Service. Participation was voluntary and written, informed consent was obtained from all participants and we followed the World Health Organisation guidelines to ensure the safety of participants.^17^ A clinical psychologist conducted most of the interviews and survivors chose a time and location convenient to them. All identifying information was removed from the data in order to protect the identity of participants.

#### Funding statement

This study was funded by Centers for Disease Control under the Cooperative Agreement U2G/PS001137.

## Results

### Survivors’ emotional struggles

All survivors interviewed experienced emotional difficulties, stigmatisation, anxiety, blame, frustrations and anger. Emotional difficulties arose from many different sources other than the rape event. Women described internal stigma that arose from using rape services (stigma of rape) and taking HIV drugs (HIV-related stigma). The initial reporting process was a source of frustration for some. A woman who finally attended many counselling sessions at an NGO explained the source of her anger when she had to repeat the statement of the incident. She said:

‘and the more I wanted to forget about it then I must speak every time. It was very sore for me because I repeatedly have to tell what happened. The night before at the police station I had to give my statement and then the detective came to me and I had to tell him the story. I tell the doctor the story. And then I had to tell the other detective again. So it was hard for me.’ (Survivor 4)

A woman who worked in health services was concerned about attending the service and said:

‘I was thinking at work they know what facilities are there in Thuthuzela and they will think what was this person doing in that centre.’ (Survivor 2)

The same woman demonstrated concerns of being stigmatised when she spoke about adherence to PEP:

‘I’m self-conscious. I can’t, when I’m around people I’m thinking maybe they will find out that I was using ARVs.’ (Survivor 2)

Another woman showed much self-blame and associated shame:

‘It feels like everybody knows. Even a stranger, when I walk past or when I walk in the road … I have a tendency to look down when I walk.’ (Survivor 10)

Despite attending counselling, she had considered suicide. Internalised stigma also arose from blame with a woman struggling with the contradiction of being told in counselling not to blame herself but people in her community telling her differently. Fear of the unknown and not getting information was also a source of anxiety. A woman explained that the detective:

‘never told me what was going to happen. He just told me he was going to take me to the hospital, I need to see one of the doctors there. Nothing else … I was scared because he had this kit with him. This rape kit. I was just watching this box the whole time. I didn’t know what was in there.’ (Survivor 4)

Women also anticipated stress related to the blame and accompanying stigmatisation from family.

### Coping

While all the survivors who were interviewed experienced emotional difficulties, stigmatisation, anxiety and anger, they presented varying levels of coping and distress during the interviews. Those who were coping well had good social support from either family members or close friends, whereas those who were struggling with hypervigilance, emotional dysregulation or flat affect, described high degrees of self-blame and stigmatisation and did not have an active support system or had not disclosed the rape to people close to them. The latter group was also more likely to report disruptions in daily functioning including insomnia, missing work, flashbacks, not trusting people, not leaving home or, in a few cases, suicidal ideation.

Some survivors recognised they were not coping. ‘I cannot really say I’m coping because, um … My family is in Pretoria and I’m new in Cape Town … so I don’t have friends’. Another woman recognised her vulnerability when not coping and said:

‘I get angry a lot, just shout at everybody at home. My mom, the niece and children, I just shout at them for no reason. I just lose it sometimes. And when I think that I’m okay, there’s a part that’s lying to myself.’ (Survivor 10)

Women used various strategies to cope but not all worked. A woman who had not followed up on her referral for counselling indicated she coped but at the same time reported hyposomnia and recurrent flashbacks. She said:

‘I don’t think more of it now. Once I start to think about it, I’ll focus my mind on something else. But every day it does come back, everyday it comes back. I make sure that I do something to forget about it.’

Another said:

‘you build up a little wall to emotionally cope with everything.’ (Survivor 8)

Others explained they coped by not telling others. A woman explained her decision not to tell her family with whom she lives:

‘Its going to come when I’m ready to tell. Because it’s too painful now. When I’m going to tell another one, that people is going to take me down that stress is going to be high you see?’ (Survivor 11)

A few women said they coped through their *faith* in God but most reported coping because of the perception of being *lucky* to be alive and:

‘knowing that this is something that could happen to anyone.’ (Survivor 7)

Or:

‘this happens to a lot of people so I figured that I am not first person it has happened to and that helped me a lot.’ (Survivor 13)

This concept was often part of the support given by family and friends (including counsellors). Another survivor said she coped as:

‘this is not the end of life.’ (Survivor 5)

Struggling to cope was also underpinned by the need to be *strong* to take the HIV prevention drugs. A woman said:

‘I knew that this is a medicine I have to take because this guy put sperms on me. So I was thinking of, uh, pregnancy and HIV.’ (Survivor 2)

### Adherence to HIV prevention medication

Of the 12 women who were given PEP, 1 stopped because of intermittent use, 3 were still taking it at the time of the interviews and the rest reported having completed the 28-day course. The woman whose poor adherence resulted in discontinuation of the drugs was confused about why she was not given additional medications at her follow-up appointment and did not understand it was because of her poor adherence. Most of the survivors reported side effects and said taking PEP medications is:

‘not easy because sometimes [*the medications*] reminds me about why I’m taking it.’ (Survivor 6)

This internal stigma related to taking HIV medication was a barrier most women appeared to manage.

### Support and care received at rape services

In general, survivors expressed gratitude for the post-rape care services they obtained:

‘I think it was helpful because of the medication and the bit of counselling that they gave.’ (Survivor 6)

Indeed, even small gestures of caring were greatly appreciated:

‘[*The doctor*] was very respectful. I really appreciated the doctor. Like, she even made some informal conversation.’ (Survivor 8)

Survivors also reported frustrations. A survivor who used a rural service described the post-rape care services as ‘a very unrehearsed play’ (Survivor 8) and lamented that in her state of shock and anxiety she would have appreciated clearer and repeated communication about the procedures. Another described feeling isolated while sitting alone in the waiting room and seeing clinic staff:

‘chatting … and laughing and stuff’ making her think they ‘don’t care’ about their work’. (Survivor 10)

Several survivors noted that they would have appreciated staff chatting to them during the wait to help them feel more comfortable and avoid flashbacks of the rape. Several survivors were also frustrated by long waiting times at the post-rape care clinic and at police stations and noted that sessions with the counsellors felt rushed at times. This was also observed by the researchers during their time spent at the clinic.

### Locating mental health support in acute, post-rape care

**FIGURE 1:**
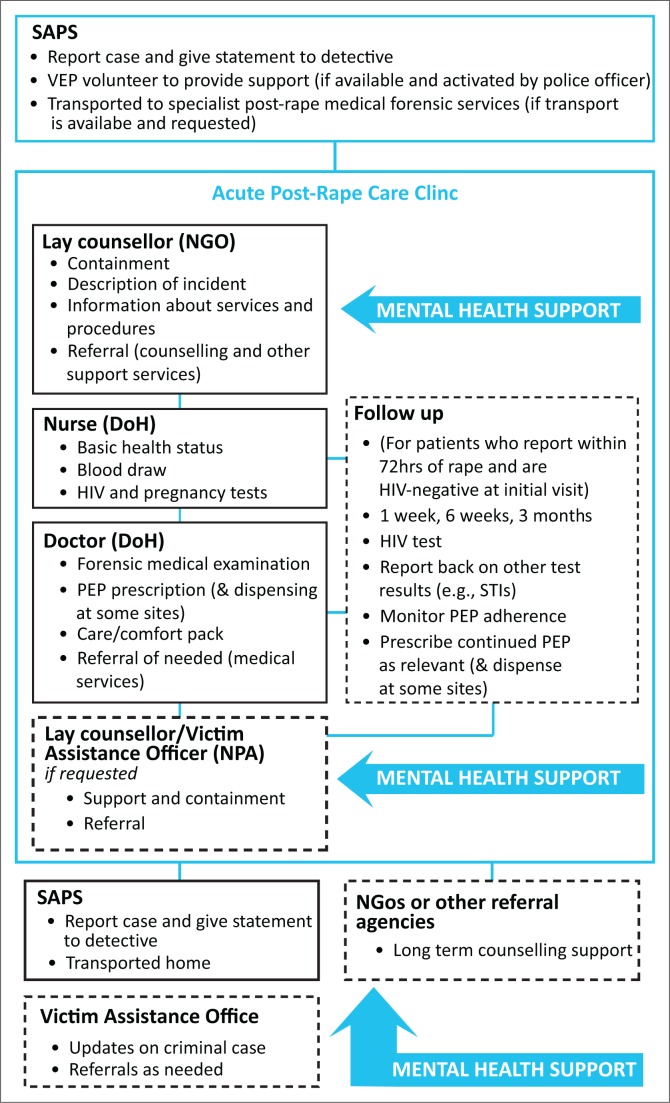
Diagram of the description of services from service providers interviewed and from observations.

Figure 1 provides an overview of the services and the location of mental health support, and it is clear mental health support is not well integrated through acute services and is entirely provided by NGOs, some of which are on a voluntary basis. At the initial visit, survivors usually first meet with a lay counsellor for 10–45 minutes and these sessions focus on ‘contain[*ing*] their feelings’ (Provider 14) and providing some information about the services with a strong focus on the medical forensic examination. Indeed, very little information on the mental health impact of rape and the typical symptoms of a trauma reaction are regularly provided. Providers explain that such information was provided in long-term counselling. Only one, very experienced counsellor described that she would explore survivors’ feelings of guilt and victim blaming experiences. During observations of counselling sessions, many counsellors only provided cursory information about the medical procedures, expressed their sympathy and often focus on asking the survivor to recount the rape incident because:

‘everybody wants the story from the horse’s mouth … I must ask my questions … I need the story so that I know what’s happening.’ (Provider 31)

Although this questioning was common practice, several providers wondered whether it may harm survivors. The counsellor’s interaction with survivors usually ended at this stage, unless survivors requested a follow-up appointment.

The DoH and NPA staff all maintained that they had little or no role in providing mental health support to survivors. The Victim Assistance Officers saw their role as providing referrals for mental health services, usually to NGOs located conveniently close to the survivor’s residence and these were usually in the form of a letter with little accompanying information, assistance in planning or follow-up if survivors did attend. One survivor described the referral she received to access a counselling NGO:

‘I don’t know what is going to happen there. I don’t know what they are doing … I still have the letter, I never opened it.’ (Survivor 11)

Few referrals were made to psychologists or psychiatrists, with providers indicating that they did not make such referrals because of the long wait to get an appointment. Indeed, this attitude and the reality of few available mental health services meant that some providers avoided probing mental health issues:

‘sometimes you hold back … because you can’t guarantee services … sometimes you’re even afraid to suggest it in case you’re opening up a can of worms. So you have to be a bit careful around that, it’s a bit constrained because of the system.’ (Provider 12)

The poor linkage to mental healthcare is evident with less than half of the survivors interviewed having accessed counselling services to which they were referred. However, the survivors who did take up counselling services noted that it was very helpful and had a positive impact on their well-being and recovery. It would appear many of the women are assisted in preparation of the court procedures. A woman explained that the counsellor provided information about ‘my thoughts and feelings … she explains to me what’s going to happen at court …’ (Survivor 4). Barriers to accessing such counselling or attending the recommended ongoing counselling sessions included lack of transport, difficulty getting time off work, poor knowledge of what to expect and perceived stigma.

### Staff capacity to provide mental health support

Although all service providers in acute post-rape care noted their concern about survivors’ mental health, few had any formal mental health training and it is not surprising that mental health literacy was generally very low. Indeed, during interviews, most providers assumed that mental health only referred to ‘mentally ill’ or ‘mentally challenged’ survivors who were described as having a ‘malfunctioning mind [*or*] a missing link’ (Provider 3). Most providers believed they had little or no impact on or role in survivors’ mental health as they were more concerned about physical health and forensic needs during the acute stage and few considered any links between survivors’ mental health and PEP adherence or service uptake. Among the service providers, lay counsellors had a fair understanding of typical mental health issues post rape describing survivors as ‘suicidal, depressed, traumatised’ and saw their role to ‘support clients to accept and move on with their lives in a healthy way’ (Provider 2). Indeed, lay counsellors had a clear role in providing mental health support to survivors, and, concurrently, other providers did not address or consider mental health support issues in their roles resulting in poor integration of mental health throughout acute post-rape care. Furthermore, mental health was not treated as a priority, with one centre going so far as to cut short, delay or skip sessions with lay counsellors. The medical provider described such sessions as:

‘tedious’ and ‘prolong[*ed*].’ (Provider 9)

Another provider noted that:

‘the mental health aspect [*should be addressed*] much later.’ (Provider 12)

Nevertheless, one doctor emphasised the need for all acute post-rape care service providers to be trained in post-rape mental health issues in order to better understand and then integrate mental health support into the service package. At the time of the study, there were no formal, validated clinical tools to assess survivors’ mental health status, despite the call of:

‘I think actually every patient should have … psychological screenings … I think it should just be part of a protocol, but it’s not.’ (Provider 12)

Lay counsellors had little power or influence to educate or influence the acute post-rape service structure or service providers and so views such as, there not being enough time to address mental health support (Provider 12) or of some survivors not ‘want[*ing*] to be counselled’ and rather ‘just actually want to go home’ (Provider 9), are accepted and perpetuate the status quo.

### Impact on service providers

Not only are service providers poorly equipped to provide mental health support to survivors, they are themselves often struggling with the stress and trauma of their work even if they find it to be ‘fulfilling’ or a ‘privilege’ (Provider 4). The workload that service providers carry is extremely high and always urgent, and the nature of the work is ‘very draining’ (Provider 7) and it ‘touches you; we are people too’ (Provider 1). Continuous exposure to rape narratives and sequelae, dealing with victim blame attitudes in the community and feeling helpless take their toll on providers: ‘you can become very depressed sometimes’ (Provider 7) or ‘you just get so angry’ (Provider 8) or it ‘take[*s*] out so much of yourself’ (Provider 2) and that it is ‘so heart sore’ (Provider 7). Providers went on to describe experiencing ‘burnout’ (Provider 2):

‘Where you reach your top. You get stressed easily. You pop up easily. Your nerves, you know, they are not stable. You’ve given and you’ve given and you’ve given until you can’t give anymore.’ Another provider described that ‘you feel like all your breath has been removed from you, you cannot breathe anywhere anymore because of the things that you’ve heard.’ (Provider 3)

Some providers described psychosomatic complaints:

‘this thing is working in your mind all the time and you will develop a headache and you will feel miserable and I don’t think that you will be able to help your next customer.’ (Provider 4)

and your:

‘body is telling [*you*] now that [*you*] are tired now … don’t want to do anything really.’ (Provider 14)

In addition, survivors described typical post-traumatic stress symptoms: feeling afraid all the time; becoming controlling and strict with their children; experiencing visual flashbacks; hyper-vigilance and exaggerated startle response; and trouble focusing on continued work tasks. Furthermore, there was some suggestion of compassion fatigue in the observed sessions when many providers maintained a disinterested, business-like demeanour with significantly limited compassion or connectedness with their patients and colleagues. There was very little formal, regular support offered to service providers other than lay counsellors receiving support to deal with work-related stress and vicarious trauma from their organisations.

## Discussion

The study has shown rape survivors’ varying levels of emotional distress post rape, which is consistent with the trauma responses post rape.^1,2,9,10^ The study confirms that receiving support and care from others helped survivors cope better,^18^ but not all were receiving it in the immediate period after the rape demonstrating the poor linkage to mental healthcare. Providing mental healthcare and support during the initial services is essential to begin the process of recovery and building adaptive coping and resilience methods among survivors.^18^ Poor response and attention to mental health needs in the immediate post-rape services and on an ongoing basis can have a detrimental impact particularly if the survivor has to endure court processes that are often very stressful and thus may exacerbate existing mental health struggles or trigger a relapse of psychological trauma. In addition, poor attention in mental health post rape may have a cascading detrimental effect on survivors’ long-term health because mental health factors are hypothesised to be critical mediating factors in HIV acquisition,^19^ and emerging studies are starting to show the associations between chronic conditions such as hypertension and diabetes with intimate partner violence.^20^

Therefore, it is a concern to find mental healthcare not prioritised or well integrated into the care provided in the immediate period post rape with the current services focused on forensic examination, biomedical intervention and legal advocacy. It is also concerning to have observed mental health support as a marginalised add-on service largely provided by NGOs who rely on donor funding, which further contributes to instability of an essential service in post-rape care. A recent report on emotional support in TCC services showed similar findings where mental health support is not prioritised and under-valued.^12^ Similar to our findings, this report showed that acute-stage services mainly include initial containment while long-term psychosocial support was seldom provided. Current mental health support was found to be an information gathering and containment session rather than a counselling session focused on psycho-education and emotional support. Poor integration also resulted in many missed opportunities and of concern is the lack of routine mental healthcare at follow-up sessions and the poor linkages to ongoing mental healthcare. Given that the survivors who did access long-term counselling at local NGOs found it beneficial, it is critical to help facilitate survivors accessing such services. Referral process should be improved to better facilitate the transition between and access to longer term support services and monitoring such referrals will also assist.

Although this study did not explore in detail the content of the initial counselling received, many accounts of the sessions, reported by both service providers and survivors and from observations, referred to elements of psychological debriefing such as a short single session, a recount of the event, information provision on expected emotional responses and reassurance of the normalcy of these emotions. This is a concern given the continued body of research that shows psychological debriefing has no benefits and may even be harmful.^21,22,23,24^ Adopting evidence-based service models for acute and long-term mental health services for rape survivors is critical but the development of effective interventions has not been a priority research focus.^25^ A promising intervention includes psychological first aid^25,26^ while ideas such as a buddy system similar to the one used in the HIV-antiretroviral therapy (ART) services may be helpful and feasible to integrate into the busy acute post-rape care services. Interventions will need to be brief in order to fit into an already highly burdened service system and also to accommodate many survivors’ struggles to access transport and secure time off from work. Cognitive Behaviour Therapy (CBT)^27^ has been shown as an effective intervention for PTSD post rape, but limited research have been done outside of developed settings. The most promising research is emerging from conflict settings where adaptation of CBT have shown promise with hope that such interventions are not impossible in poor resource settings with high prevalence of rape.^28^

Another challenge is the low levels of mental health literacy and capacity among service providers. This may have contributed to the poor response and recognition of survivor’s mental health needs as well as an understanding of how their interactions impact on survivors’ mental health. Providers’ capacity to respond to survivors may have been linked to their own mental health and ability to cope as this study found a strong emerging theme of service providers’ mental health and well-being challenges. Providers revealed post-rape services were very stressful because of a combination of high workload, the traumatic nature of the work and little or no support for their mental health and well-being. Several providers discussed struggling with vicarious trauma reactions and compassion fatigue that negatively affected their work and personal lives. This is well documented in the literature,^29^ and if providers better understood their own mental health and vicarious trauma and were better supported in self-care and regular support or debriefing activities, then they are more likely to pass on some of this knowledge and attitudes to survivors. Furthermore, their capacity for relating to and engaging with clients in an ongoing compassionate and empathic way is likely to be enhanced, because they will not resort to the self-protective strategies of detachment and distress intolerance that were witnessed in this study. Therefore, it is critical for all staff working within this service to be given foundational pre-service and ongoing in-service training in trauma-related mental health as this will reduce mental health illiteracy, vicarious trauma and compassion fatigue. Such addition to staff support is not expected to be hugely resourceful as it will only require adjustments within the current training package.

A limitation of the study is that it was a rapid assessment of mental health support in the immediate period post rape and it was conducted in the Western Cape Province only. We do not know the state of mental health services in other provinces, but a recent report from Vetten shows similar findings.^12^ Another limitation is that we did not engage with policy makers or review post-rape care policies and we are not able to comment on the extent to which policy makers and policies acknowledge and address mental health within acute post-rape services.

## Conclusion

Although great strides in providing acute post-rape care within a comprehensive or ‘one-stop’ specialist service has been achieved, such a service cannot be considered truly comprehensive without providing essential high-quality mental health support and care for both survivors and service providers. Improving the general approach and manner of engaging survivors and strengthening the linkages to ongoing mental healthcare are essential in order to better integrate mental health services into the post-rape care service package.
